# Draft Genome Sequence of a Community-Acquired Methicillin-Resistant *Staphylococcus aureus* USA300 Isolate from a River Sample

**DOI:** 10.1128/genomeA.01166-17

**Published:** 2017-10-19

**Authors:** Sarah Lepuschitz, Robert Mach, Burkhard Springer, Franz Allerberger, Werner Ruppitsch

**Affiliations:** a Institute of Medical Microbiology and Hygiene, Austrian Agency for Health and Food Safety, Graz, Austria; b Vienna University of Technology, Research Area of Biochemical Technology, Institute of Chemical, Biological and Environmental Engineering, Vienna, Austria; c Department of Biotechnology, University of Natural Resources and Life Sciences, Vienna, Austria

## Abstract

The increasing emergence of multiresistant bacteria in health care settings in the community and in the environment represents a major health threat worldwide. Here, we report the draft genome sequence of a community-acquired methicillin-resistant *Staphylococcus aureus* (CA-MRSA) USA300 isolate (W1) from a small river in southern Austria.

## GENOME ANNOUNCEMENT

In recent years, the rising evolution of antibiotic-resistant bacteria (ARB) and antibiotic resistance genes (ARGs) has been prompted by the release of antibiotics as pollutants in aquatic environments (1). There, ARGs are able to persist and even spread through horizontal gene transfer, genetic mutation, and recombination of ARB (2). The dissemination of increasing antibiotic-resistant human pathogens, such as methicillin-resistant *Staphylococcus aureus* (MRSA), is a growing public health threat; therefore, it is of major importance to understand the circumstances which enhance the evolution and distribution of ARGs (3).

In 2016, the Austrian Agency for Health and Food Safety started a pilot project to investigate antimicrobial resistance (AMR) in surface water. Among several water samples from diverse Austrian rivers, one sample from a small river in the province Carinthia contained a MRSA isolate. Whole-genome sequence analysis of this water isolate, W1, identified the main characteristics of community-acquired MRSA (CA-MRSA) USA300: sequence type 8 (ST8), *spa* type t008, staphylococcal cassette chromosome *mec* element type IV (SCC*mec* IV), Panton-Valentine leukocidin (PVL), and the arginine catabolic mobile element (ACME) cluster. This is the first report and draft genome sequence of a MRSA USA300 isolate derived from a water sample.

For the isolation of *S. aureus* from water samples, 100-ml aliquots were filtrated, and filters were incubated in thioglycolate at 37°C overnight. For the detection of MRSA, the overnight culture was plated on chromogenic medium (BBL CHROMagar MRSA II; Becton, Dickinson, Vienna, Austria). The water isolate was further identified as *Staphylococcus aureus* using matrix-assisted laser desorption ionization–time of flight (MALDI-TOF) mass spectrometry (Bruker, Billerica, MA). High-quality genomic DNA from an overnight culture was obtained using the MagAttract high-molecular-weight (HMW) DNA kit (Qiagen, Hilden, Germany). The quantification of input DNA was carried out with a Qubit 2.0 fluorometer (Thermo Fisher Scientific, Waltham, MA, USA) using the double-stranded DNA (dsDNA) BR assay kit (Thermo Fisher Scientific). Library preparation for whole-genome sequencing was done with a Nextera XT kit (Illumina, Inc., San Diego, CA, USA), according to the manufacturer’s protocol. Paired-end sequencing (2 × 300 bp) was performed on a MiSeq instrument (Illumina, Inc.) and generated 557,130 reads from 142,372,680 unassembled nucleotides. For assembly into a draft genome, raw reads were *de novo* assembled using SPAdes version 3.9.0 (4). Contigs were filtered for a minimum coverage of 5-fold and minimum length of 200 bp, which resulted in 380 contigs with a total of 3,016,290 nucleotides at a coverage of 50-fold. The NCBI Prokaryotic Genome Automatic Annotation Pipeline identified 3,348 genes, 3,267 coding sequences, 118 pseudogenes, 21 rRNA operons (9 complete, 12 partial), and 56 tRNA genes.

### Accession number(s).

This whole-genome shotgun project has been deposited at DDBJ/ENA/GenBank under the accession number NKCX00000000. The version described in this paper is version NKCX01000000.

## References

[1] Xu Y, Guo C, Luo Y, Lv J, Zhang Y, Lin H, Wang L, Xu J 2016 Occurrence and distribution of antibiotics, antibiotic resistance genes in the urban rivers in Beijing, China. Environ Pollut 213:833–840. doi:10.1016/j.envpol.2016.03.054. 27038570

[2] Berendonk TU, Manaia CM, Merlin C, Fatta-Kassinos D, Cytryn E, Walsh F, Bürgmann H, Sørum H, Norström M, Pons MN, Kreuzinger N, Huovinen P, Stefani S, Schwartz T, Kisand V, Baquero F, Martinez JL 2015 Tackling antibiotic resistance: the environmental framework. Nat Rev Microbiol 13:310–317. doi:10.1038/nrmicro3439. 25817583

[3] Allen HK, Donato J, Wang HH, Cloud-Hansen KA, Davies J, Handelsman J 2010 Call of the wild: antibiotic resistance genes in natural environments. Nat Rev Microbiol 8:251–259. doi:10.1038/nrmicro2312. 20190823

[4] Nurk S, Bankevich A, Antipov D, Gurevich AA, Korobeynikov A, Lapidus A, Prjibelski AD, Pyshkin A, Sirotkin A, Sirotkin Y, Stepanauskas R, Clingenpeel SR, Woyke T, McLean JS, Lasken R, Tesler G, Alekseyev MA, Pevzner PA 2013 Assembling genomes and mini-metagenomes from chimeric MDA products. J Comput Biol 20:714–737. doi:10.1089/cmb.2013.0084. 24093227 PMC3791033

